# Application of 4-way decomposition to the analysis of placental-fetal biomarkers as intermediary variables between maternal body mass index and birthweight

**DOI:** 10.3389/frph.2022.994436

**Published:** 2022-12-05

**Authors:** Xiaoshuang Xun, Xu Qin, Alexander J. Layden, Qing Yin, Shanna H. Swan, Emily S. Barrett, Nicole R. Bush, Sheela Sathyanarayana, Jennifer J. Adibi

**Affiliations:** ^1^ Department of Epidemiology, School of Public Health, University of Pittsburgh, Pittsburgh, PA, United States; ^2^ Department of Health and Human Development, School of Education, University of Pittsburgh, Pittsburgh, PA, United States; ^3^ Department of Biostatistics, School of Public Health, University of Pittsburgh, Pittsburgh, PA, United States; ^4^ Department of Environmental Medicine and Public Health, Icahn School of Medicine at Mount Sinai, New York, NY, United States; ^5^ Department of Biostatistics and Epidemiology, Rutgers School of Public Health, Rutgers University, Piscataway, NJ, United States; ^6^ Department of Psychiatry, University of California San Francisco, San Francisco, CA, United States; ^7^ Departmetn of Pediatrics, University of Washington, Seattle, WA, United States; ^8^ Department of Obstetrics, Gynecology and Reproductive Sciences, University of Pittsburgh, Pittsburgh, PA, United States

**Keywords:** birthweight, maternal BMI, placental hormones, causal mediation analysis, reproductive epidemiology

## Abstract

Human chorionic gonadotropin (hCG) is a placental hormone measured in pregnancy to predict individual level risk of fetal aneuploidy and other complications; yet may be useful in understanding placental origins of child development more generally. hCG was associated with maternal body mass index (BMI) and with birthweight. The primary aim here was to evaluate hCG as a mediator of maternal BMI effects on birthweight by causal mediation analysis. Subjects were 356 women from 3 U.S. sites (2010–2013). The 4-way decomposition method using med4way (STATA) was applied to screen for 5 types of effects of first trimester maternal BMI on birthweight: the total effect, the direct effect, mediation by hCG, additive interaction of BMI and hCG, and mediation in the presence of an additive interaction. Effect modification by fetal sex was evaluated, and a sensitivity analysis was performed to evaluate the assumption of unmeasured confounding. Additional placental-fetal biomarkers [pregnancy associated plasma protein A (PAPPA), second trimester hCG, inhibin-A, estriol, alpha fetoprotein] were analyzed for comparison. For first trimester hCG, there was a 0.20 standard deviation increase in birthweight at the 75th vs. 25th percentile of maternal BMI (95% CI 0.04, 0.36). Once stratified, the direct effect association was null in women carrying females. In women carrying males, hCG did not mediate the relationship. In women carrying females, there was a mediated effect of maternal BMI on birthweight by hCG in the reverse direction (−0.06, 95% CI: −0.12, 0.01), and a mediated interaction in the positive direction (0.06, 95% CI 0.00, 0.13). In women carrying males, the maternal BMI effect on birthweight was reverse mediated by PAPPA (−0.09, 95% CI: −0.17, 0.00). Sex-specific mediation was mostly present in the first trimester. Second trimester AFP was a positive mediator of maternal BMI effects in male infants only (0.06, 95% CI: −0.01, 0.13). Effect estimates were robust to potential bias due to unmeasured confounders. These findings motivate research to consider first trimester placental biomarkers and sex-specific mechanisms when quantifying the effects of maternal adiposity on fetal growth.

## Introduction

Birthweight is a standard measure reflective of intrauterine growth and an important determinant of the life-long health of the fetus. Both low (<2,500 g) and high birthweight (>4,000 g) are associated with an increased risk of hypertension, diabetes, and kidney disease during childhood or later life (1, 2). Maternal body mass index (BMI) and fetal sex are two established determinants of birthweight. Higher pre-pregnancy BMI is associated with a higher birthweight and higher fat mass in neonates, possibly due to excess nutrition transmitted to fetuses (3, 4). Boys are approximately 130 grams heavier at birth than girls after normalizing for gestational age at delivery (5). The positive association between maternal BMI and birthweight is stronger in male vs. female infants (6).

Human chorionic gonadotropin (hCG) is a glycoprotein hormone produced by the placenta that supports fetal growth and development by regulating onset of fetal steroidogenesis and sex differentiation in the male among other functions (7–9). hCG is clinically used for screening of fetal aneuploidies, and holds potential for application in epidemiologic research on the developmental origins of health and disease (10). hCG is associated with birthweight and with sex-specific fetal growth (11, 12). hCG levels vary by fetal sex (13–16).

Maternal BMI is negatively associated with hCG during early pregnancy (17, 18). As maternal BMI can alter hCG levels, and hCG levels during pregnancy are related to birthweight, we hypothesize that hCG could be an intermediate variable in the association between maternal BMI and birthweight. Fetal sex may operate as an effect modifier of single associations or the overall pathway. We studied BMI in the trimester before appreciable pregnancy-related change in BMI has occurred, and when fetal cells are programmed and are susceptible to exposures (19, 20).

We cannot *a priori* predict if hCG, plays a role as a mediator, a modifier, or both in the mechanism underlying the association of maternal BMI on birthweight. Therefore, we applied the 4-way decomposition that was originally proposed by Vanderweele to decompose the total effect into four components (21). The four components represent the portions of the total effect due to (1) mediation but not interaction, (2) interaction but not mediation, (3) both mediation and interaction, and (4) neither mediation nor interaction.

The first aim of the analysis was to gain insight into the role of hCG as a potential biologically and statistically meaningful intermediary variable that can improve estimation of the effects of maternal BMI in pregnancy. A second aim was to evaluate and illustrate the application of the 4-way decomposition using the STATA med4way package (22). A third aim was to conduct the same analysis using the full set of placental-fetal biomarkers available through the prenatal medical record namely, pregnancy associated plasma protein A (PAPPA) measured in the first trimester, and hCG, inhibin-A, estriol, and alpha fetoprotein (AFP) measured in the second trimester.

## Methods

### Study population

From 2010 to 2013, 753 women at university-based prenatal clinics in 4 U.S. urban sites (San Francisco, CA; Seattle, WA; Minneapolis, MN; Rochester, NY) were recruited into The Infant Development and Environment Study (TIDES) during late first trimester as previously described (23). Briefly, a woman was eligible if she was >18 years of age, able to read and write in English, planned to deliver in an eligible hospital, and provided signed informed consent. All mothers gave birth to live singletons. This analysis includes all TIDES pregnancies that had a first and/or second trimester prenatal serum screening. The UW clinical site did not measure first trimester hCG.

### Data collection

Questionnaires were completed by subjects in each trimester to provide information on demographics, reproductive history and health, lifestyle, and stressful life events (24). Birthweight, infant sex, and gestational age at delivery were abstracted from the birth record. First trimester maternal BMI was calculated as weight (kg) divided by squared height (m^2^) based on self-report of current weight and height in the first trimester questionnaire. The first trimester questionnaire was completed and the blood draw occurred at the same visit (25).

### Mediator: placental-fetal hormone measurements

hCG was measured as part of hospital-based prenatal serum screening programs for Down's Syndrome and other aneuploidies (26). Total hCG (intact hCG which includes the *α* and *β*-subunits) and/or hCG-*β* were measured depending on which laboratory did the analysis. When measured together, serum intact hCG and hCG-*β* are correlated with *R* value = 0.96) and therefore were treated as equivalent measures (27). Data on hCG and the other placental-fetal biomarkers (first trimester PAPPA, second trimester hCG, Inhibin-A, Estriol, and Alphafeto protein) were abstracted from the prenatal medical record or obtained directly from the laboratories that conducted the analyses. UCSF data were obtained from the California Genetic Disease Screening Program, and all analytes were measured by the AutoDELFIA time-resolved fluoroimmunoassay (PerkinElmer). At the other sites, samples were analyzed in University or private laboratories. For subjects at UW and UMN, analytes were measured by UniCel DxI 800 Immunoassay System (Beckman Coulter). For subjects at URMC, analytes were measured by Uni- Cel DxI 600 Immunoassay System (Beckman Coulter). Due to small differences of assays and reporting units, biomarker concentrations were transformed into *z*-scores to standardize individual values to the mean and standard deviation (SD) of each site. This allowed for clinically derived data to be merged across the TIDES sites. Mean gestational age of serum collection was 12 weeks (SD = 1 week, range = 10–14 weeks) for first trimester, and 17 weeks (SD = 2, range = 15–20 weeks) for second trimester. We did not further adjust for gestational age at serum sample collection, as it does not contribute to birthweight.

### Outcome: birthweight

Birthweight (kg) was obtained from birth records. Birthweight *z*-scores were used to normalize for gestational age at delivery and fetal sex using US population-based references as fetal sex and gestational age at birth are important sources of variability in birthweight but are not causes of first trimester maternal BMI (28).

## Statistical analysis

Baseline characteristics of the study sample were compared between women carrying male and female fetuses using two-sample t-test and chi-square test. Linear regression was used to estimate associations between (1) maternal first trimester BMI and birthweight (exposure-outcome); (2) BMI and hCG levels (exposure-mediator); and (3) hCG levels and birthweight (mediator-outcome). All models included an interaction of fetal sex.

The decomposition approach requires that the exposure be treated as a dichotomous exposure. We contrasted the 75th percentile of BMI vs. the 25th percentile, which fell into overweight (BMI 25 to <30 kg/m^2^) and normal weight (18.5 to <25 kg/m^2^) categories. First trimester and pre-pregnancy BMI are highly correlated, and hence we applied non-pregnant standard cut-offs for overweight and normal weight (29).

The 4-way decomposition analysis was used to calculate and decompose the total effect (TE) into four distinct quantities. The first quantity is the pure indirect effect (PIE) solely transmitted through hCG. The second quantity is the reference interaction between BMI and hCG, which does not operate by BMI changing the mediator. The third quantity is the mediated interaction, which requires that BMI changes hCG while interacting with hCG in affecting the outcome. The fourth quantity is the controlled direct effect (CDE), which is the effect of first trimester BMI on birthweight when hCG levels for all women are set to a single value. In our case, we set hCG to the mean value. This assumes artificially that all women are at the “normal value” of hCG, allowing for the estimation of the direct effect that is not biased by the intermediate pathway. Details on these different quantities have been described previously and are included in Supplementary Table S6 (30). The software generates estimates of the proportion of the total effect for each of the 4 indirect effects. The proportions can be negative or positive and add up to 100%.

The decomposition analysis was conducted in the full data set, and in data sets stratified by sex. In the models stratified by fetal sex, BMI cut points and hCG mean values were re-calculated for women carrying females and males separately, to allow for different underlying distributions and mechanisms by sex. The above steps were repeated for first trimester PAPPA, and second trimester hCG, Estriol, Inhibin-A, and AFP.

**Figure 1:**
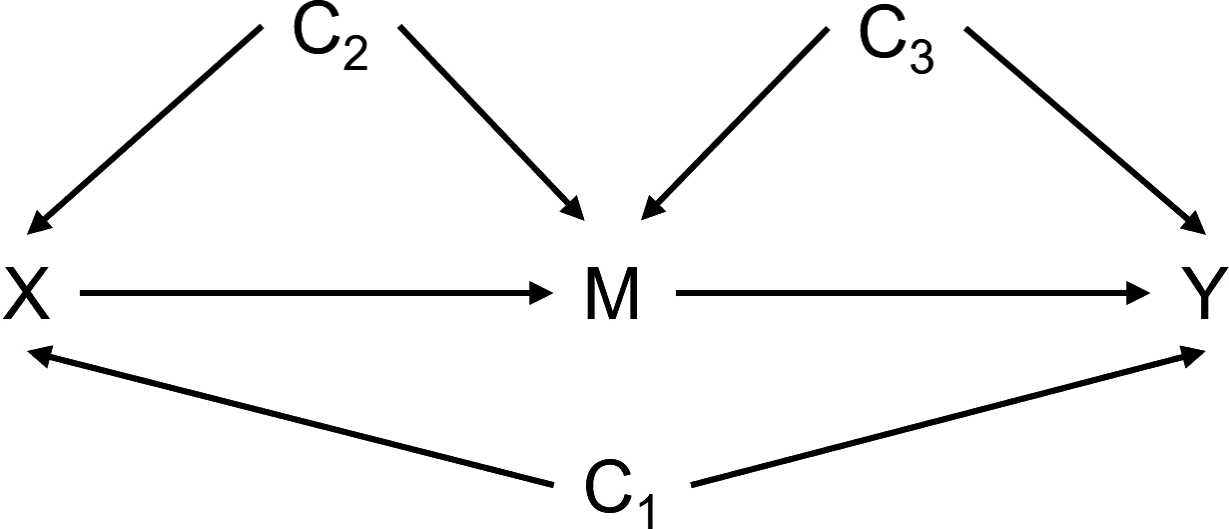
Directed acyclic graph (DAG) for the impact of maternal first trimester BMI on birthweight when treating placental-fetal biomarkers as an intermediary variable. X, exposure – maternal first trimester body mass index; M, mediator – maternal serum placental-fetal biomarkers; Y, outcome – birthweight z-score; C_1_, confounders of X-Y, the BMI and birthweight association; C_2_, confounders of X-M, the BMI and placental-fetal biomarker association; C_3_, confounders of M-Y, the placental-fetal biomarker and birthweight association The med4way macro does not distinguish between the C_1_, C_2_, C_3_ sets.

A directed acyclic graph (DAG) was used to identify sources of confounding between exposure-outcome, exposure-mediator, and mediator-outcome (31) (Figure 1). All models were adjusted for TIDES center, parity status (0, 1, >1), marital status (Partnered, Single/Previously Married), maternal age, race (White, Black, Asian, Other/Mixed), income level (<$25,000, $25,000-$74,999, ≥$75,000), education level (High school or less, Some college, College graduate, and Graduate School), smoking status (Current/Ever, Never), and first trimester stressful life events (None, Any). Maternal urinary phthalates, putative obesogenic chemicals, were evaluated as confounders and not included in the final models (23, 32). For 356 subjects, 9 women (2.5%) had missing data of education level (*n* = 2), income level (*n* = 5), birthweight (*n* = 1), and first trimester BMI (*n* = 1). Therefore, data imputation was not implemented.

A bootstrapped analysis was used to empirically evaluate the difference in the mediation results between male and female infants for first trimester hCG only. We resampled the dataset 1,000 times for male and females separately, did mediation analysis in each dataset, then pooled the results together to calculate the difference and variance in the 5 decomposition quantities. Of the 1,000 estimates, we used the values at the 2.5th, 50th, and 97.5th percentiles to evaluate differences.

R (version 4.2.0; Vienna, Austria) was used for data analyses. The decomposition analysis was conducted using the STATA (version 14.2; College Station, TX) package, med4way (22).

### Sensitivity analysis

Four-way decomposition analysis relies on the assumption that conditional on the set of baseline characteristics, (1) there is no unmeasured confounding of the exposure-outcome relationship, (2) there is no unmeasured confounding of the mediator-outcome relationship, (3) there is no unmeasured confounding of the exposure-mediator relationship, and (4) there are no confounders of the mediator-outcome relationship that may be affected by the exposure (post-exposure confounders) (33). Potential unmeasured confounders include maternal diet (composition, quality, quantity, physical activity, obesogens and maternal and paternal genetics/epigenetics). A sensitivity analysis can help to determine if unmeasured pre-exposure confounding would easily change analytic conclusions. This has not been discussed in previous decomposition studies (21, 22, 30). The sensitivity of the initial results to unmeasured confounding was assessed by a two-step process (34–36). First, we assessed change in each effect estimate due to omission of each observed confounder. This provides a plausible range of bias, based on the assumption that the confounding role of an unmeasured pre-exposure confounder is comparable to that of a measured confounder. Second, we remove the bias from the original effect estimate and confidence interval (adjusted effect estimate = original estimate − bias). If the sign or the precision of the effect is changed after the adjustment, we conclude that the initial results are sensitive to unmeasured confounding.

## Results

### Study population

Descriptive characteristics of the study population (*n* = 525) are presented in Table 1, and a comparison to the full TIDES cohort (Supplementary Table S1). In the sample, 48% of pregnancies were carrying male fetuses, 45% were overweight/obese (BMI > 25 kg/m^2^ range), 67% were White, 12% were Black, 43% completed graduate school, and 80% were partnered. There was no difference in mean BMI among women carrying male and female fetuses. Male babies had a 160-gram higher mean birthweight than female babies. Women carrying female fetuses had a hCG *z*-score that was 0.21 above the mean value and women carrying male fetuses had a hCG *z*-score that was 0.25 below the mean. Women with a first trimester hCG value did not differ appreciably from the full cohort except there were slightly less women in the mid-income category and slightly more women in the high-income category (Supplementary Table S1).

**Table 1:** Demographics of study subjects in the infant development and environment study (TIDES), 2010–2013.

	Overall (*N* = 525)	Women carrying male fetus (*N* = 250)	Women carrying female fetus (*N* = 275)	*P*-value male vs. female fetus
Fetal sex, first trimester hCG, *N* (%)	356 (69)	172 (48)	184 (52)	0.71
Fetal sex, first trimester PAPP-A, *N* (%)	427 (81)	200 (47)	227 (53)	0.52
Fetal sex, second trimester hCG, *N* (%)	262 (50)	123 (47)	139 (53)	0.83
Fetal sex, second trimester Inhibin-A, *N* (%)	262 (50)	123 (47)	139 (53)	0.83
Fetal sex, second trimester Estriol, *N* (%)	262 (50)	123 (47)	139 (53)	0.83
Fetal sex, second trimester Alpha fetoprotein, *N* (%)	393 (50)	187 (48)	206 (52)	1.00
Maternal age, years, mean (SD)	31.38 (5.80)	31.12 (5.70)	31.61 (5.89)	0.34
Maternal first trimester BMI, kg/m^2^, mean (SD)	26.37 (6.32)	26.86 (6.41)	25.93 (6.21)	0.09
Birthweight, kg, mean (SD)	3.33 (0.53)	3.41 (0.55)	3.25 (0.5)	<0.01
Birthweight *z*-score, kg, mean (SD)	0.00 (1.00)	0.05 (1.05)	−0.05 (0.96)	0.27
First trimester gestational age of sample collection, weeks, mean (SD)	12.12 (0.93)	12.13 (0.92)	12.11 (0.95)	0.78
Second trimester gestational age of sample collection, weeks, mean (SD)	16.93 (1.64)	16.93 (1.80)	16.94 (1.49)	0.94
Gestational age at delivery, weeks (SD)	39.31 (1.71)	39.28 (1.70)	39.33 (1.73)	0.76
First trimester hCG *z*-score, mean (SD)	−0.01 (1.00)	−0.25 (0.89)	0.21 (1.06)	<0.01
First trimester PAPPA *z*-score, mean (SD)	−0.02 (0.98)	−0.14 (0.92)	0.08 (1.01)	0.02
Second trimester hCG *z*-score, mean (SD)	0.00 (1.00)	0.02 (1.10)	−0.03 (0.90)	0.69
Second trimester estriol *z*-score, mean (SD)	0.00 (1.00)	−0.04 (1.02)	0.04 (0.98)	0.53
Second trimester inhibin-A *z*-score, mean (SD)	0.00 (1.01)	−0.05 (0.93)	0.05 (1.07)	0.42
Second erimester alpha fetoprotein *z*-score, mean (SD)	−0.01 (1.00)	0.03 (0.95)	−0.04 (1.05)	0.51
**Race, *N* (%)**				0.91
White	350 (67)	169 (68)	181 (66)	
Black	70 (13)	35 (14)	35 (13)	
Asian	38 (7)	17 (7)	21 (8)	
Other/mixed	65 (12)	29 (12)	36 (13)	
**Study Center, *N* (%)**				0.53
UCSF, California	167 (32)	79 (32)	88 (32)	
UMN, Minnesota	101 (19)	50 (20)	51 (19)	
URMC, New York	180 (34)	90 (36)	90 (33)	
UW, Washinton	77 (15)	31 (12)	46 (17)	
**Education, *N* (%)**				0.24
High school or less	87 (17)	38 (15)	49 (18)	
Some college	72 (14)	42 (17)	30 (11)	
College graduate	141 (27)	68 (27)	73 (27)	
Graduate school	221 (42)	101 (41)	120 (44)	
**Income, *N* (%)**				0.56
<$25,000	172 (34)	81 (33)	91 (35)	
$25,000-$74,999	74 (15)	40 (16)	34 (13)	
≥$75,000	261 (51)	124 (51)	137 (52)	
**Stress events in the first trimester, *N* (%)**				0.44
None	414 (79)	193 (77)	221 (80)	
Any (≥1)	111 (21)	57 (23)	54 (20)	
**Smoking status, *N* (%)**				0.72
Not currently	478 (92)	230 (93)	248 (92)	
Current	41 (8)	18 (7)	23 (8)	
**Parity, *N* (%)**				0.36
0	176 (34)	79 (33)	97 (36)	
1	155 (30)	81 (33)	74 (28)	
>1	180 (35)	83 (34)	97 (36)	
**Marital status, *N* (%)**				0.71
Partnered	418 (80)	202 (81)	216 (79)	
Single/previously married	105 (20)	48 (19)	57 (21)	

SD, standard deviation; BMI, body mass index.

### Exposure, mediator, and outcome associations based on individual regressions

**Figure 2:**
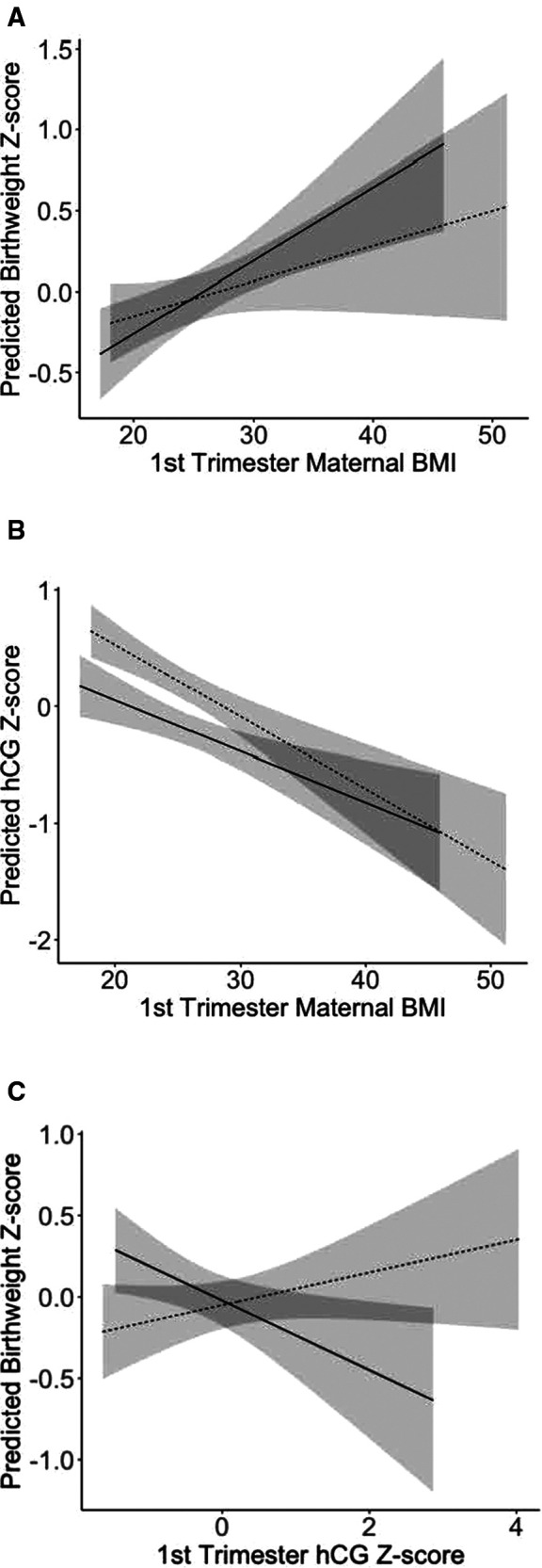
Linear associations between (**A**) maternal first trimester BMI and birthweight; (**B**) first trimester BMI and hCG; (**C**) first trimester hCG and birthweight for women carrying male and female fetuses in the infant development and environment study (TIDES), 2010–2013. Solid lines represent women carrying male fetuses, and dashed lines represent women carrying female fetuses. 95% confidence intervals are shaded around the lines. BMI, body mass index; hCG, human chorionic gonadotropin.

The associations of maternal BMI and BW were positive in both sexes, as expected from prior reports (Figure 2A). The association of maternal BMI and hCG in both sexes were negative as expected from previous reports (Figure 2B). The association of hCG and birthweight was positive in female babies and negative in male babies (Figure 2C). Fetal sex was an effect modifier of the mediator (hCG) to outcome (birthweight) (Wald test for additive interaction *P *= 0.01), and not a modifier of the associations of exposure (BMI) and mediator (hCG) (*P *= 0.30) or the exposure (BMI) and outcome (birthweight) associations (*P* = 0.20).

### Decomposition

In the analysis that combined male and female infants, the total and controlled direct effects of BMI on birthweight were all positive and similar in magnitude across the hormone subsets (Table 2). A one unit increase in BMI was associated with approximately a 20% increase in a standard deviation of the birthweight *z*-score. First trimester hCG and PAPPA were the only analytes that showed evidence of an indirect effect. Indirect effects (reference and mediated interactions) for first and second trimester hCG were almost identical, even though the second trimester sample was 25% smaller. For PAPPA, there was a weak but positive mediated interaction of 0.04 (95% CI −0.004, 0.09) and a negative pure mediation effect of −0.04 (−0.08, 0.003).

**Table 2:** Four-way decomposition of the association of maternal first trimester BMI and birthweight *z*-scores, with circulating levels of placental-fetal proteins (*z*-score) as putative mediators.

	Effect estimates (95% CI), 75th vs. 25th percentile of BMI	Proportion
**First trimester hCG**	**75th vs. 25th percentile of BMI: 21.91, 29.81**	
Total effect	0.20 (0.04, 0.36)	100%
Controlled direct effect	0.18 (0.01, 0.35)	91%
Reference interaction	−0.04 (−0.08, 0.002)	−21%
Mediated interaction	0.08 (0.01, 0.16)	42%
Pure indirect effect	−0.02 (−0.08, 0.03)	−12%
**First trimester PAPP-A**	**75th vs. 25th percentile of BMI: 21.87, 28.79**	
Total effect	0.15 (0.03, 0.28	100%
Controlled direct effect	0.18 (0.05, 0.31)	114%
Reference interaction	−0.03 (−0.06, 0.01)	−17%
Mediated interaction	0.04 (−0.004, 0.09)	28%
Pure indirect effect	−0.04 (−0.08, 0.003)	−25%
**Second trimester hCG**	**75th vs. 25th percentile of BMI: 21.79, 29.83**	
Total effect	0.23 (0.03, 0.43)	
Controlled direct effect	0.21 (0.01, 0.40)	90%
Reference interaction	−0.04 (−0.08, 0.01)	−16%
Mediated interaction	0.05 (−0.01, 0.11)	23%
Pure indirect effect	0.01 (−0.03, 0.05)	4%
**Second trimester Inhibin-A**	**75th vs. 25th percentile of BMI: 21.79, 29.83**	
Total effect	0.26 (0.07, 0.45)	100%
Controlled direct effect	0.27 (0.09, 0.46)	106%
Reference interaction	−0.01 (−0.03, 0.02)	−3%
Mediated interaction	0.01 (−0.03, 0.05)	4%
Pure indirect effect	−0.02 (−0.06, 0.02)	−7%
**Second trimester Estriol**	**75th vs. 25th percentile of BMI: 21.79, 29.83**	
Total effect	0.28 (0.10, 0.46)	100%
Controlled direct effect	0.28 (0.10, 0.46)	102%
Reference interaction	0.00 (−0.01, 0.01)	0%
Mediated interaction	0.00 (−0.01, 0.01)	1%
Pure indirect effect	−0.01 (−0.03, 0.01)	−3%
**Second trimester Alpha fetoprotein**	**75th vs. 25th percentile of BMI: 21.85, 29.99**	
Total effect	0.18 (0.04, 0.33)	100%
Controlled direct effect	0.15 (0.01, 0.29)	81%
Reference interaction	0.00 (−0.03, 0.02)	−3%
Mediated interaction	0.01 (−0.03, 0.05)	5%
Pure indirect effect	0.03 (−0.01, 0.07)	17%

In the stratified analysis of women carrying males and women carrying females, the direct and indirect effects were not consistent across trimesters. In women carrying male infants, the total effect (TE) of being at the 75th vs. the 25th percentile of BMI increased birthweight *z*-score by 0.20–0.30 units (20%–30% of a standard deviation in male birthweight). In women carrying females, the TE of maternal BMI on birthweight was null in the first trimester (Table 3). In the second trimester, the TE and CDE in females and males were similar.

**Table 3:** Four-way decomposition of the association of maternal first trimester BMI and birthweight *z*-scores, stratified by sex of the baby.

	Women carrying male fetuses	Proportion	Women carrying female fetuses	Proportion
	**First trimester hCG**			
*N*	172		184	
Total effect	0.27 (0.05, 0.50)	100%	0.08 (−0.10, 0.27)	100%
Controlled direct effect	0.23 (−0.01, 0.46)	83%	0.11 (−0.08, 0.30)	158%
Reference interaction	−0.03 (−0.08, 0.03)	−9%	−0.04 (−0.09, 0.01)	−43%
Mediated interaction	0.05 (−0.04, 0.14)	17%	0.06 (0.00, 0.13)	74%
Pure indirect effect	0.03 (−0.04, 0.10)	10%	−0.06 (−0.12, 0.01)	−89%
	**First trimester PAPP-A**			
*N*	200		227	
Total effect	0.23 (0.01, 0.45)	100%	0.10 (−0.06, 0.26)	100%
Controlled direct effect	0.29 (0.05, 0.52)	125%	0.10 (−0.06, 0.26)	104%
Reference interaction	−0.04 (−0.12, 0.04)	−18%	−0.01 (−0.04, 0.02)	−12%
Mediated interaction	0.07 (−0.07, 0.21)	31%	0.02 (−0.02, 0.06)	17%
Pure indirect effect	−0.09 (−0.17, 0.00)	−39%	−0.01 (−0.05, 0.03)	−9%
	**Second trimester hCG**			
*N*	123		139	
Total effect	0.23 (−0.08, 0.54)	100%	0.21 (0.00, 0.42)	100%
Controlled direct effect	0.17 (−0.14, 0.49)	76%	0.21 (0.00, 0.42)	100%
Reference interaction	−0.05 (−0.15, 0.04)	−24%	−0.01 (−0.04, 0.02)	−5%
Mediated interaction	0.08 (−0.05, 0.21)	35%	0.01 (−0.03, 0.05)	6%
Pure indirect effect	0.03 (−0.04, 0.10)	13%	0.00 (−0.04, 0.04)	−1%
	**Second trimester Inhibin-A**			
*N*	123		139	
Total effect	0.23 (−0.07, 0.53)	100%	0.26 (0.06, 0.47)	100%
Controlled direct effect	0.25 (−0.04, 0.55)	109%	0.28 (0.08, 0.49)	107%
Reference interaction	−0.03 (−0.08, 0.03)	−12%	0.02 (−0.02, 0.07)	9%
Mediated interaction	0.04 (−0.03, 0.11)	16%	−0.04 (−0.09, 0.02)	−14%
Pure indirect effect	−0.03 (−0.10, 0.03)	−14%	−0.01 (−0.04, 0.03)	−2%
	**Second trimester Estriol**			
*N*	123		139	
Total effect	0.30 (0.02, 0.59)	100%	0.23 (0.02, 0.43)	100%
Controlled direct effect	0.31 (0.02, 0.59)	101%	0.23 (0.03, 0.43)	101%
Reference interaction	0.00 (−0.02, 0.02)	1%	0.00 (−0.02, 0.02)	−1%
Mediated interaction	−0.01 (−0.05, 0.03)	−3%	0.00 (−0.02, 0.02)	0%
Pure indirect effect	0.01 (−0.03, 0.04)	2%	0.00 (−0.04, 0.04)	0%
	**Second trimester AFP**			
*N*	187		206	
Total effect	0.17 (−0.03, 0.37)	100%	0.21 (0.02, 0.41)	100%
Controlled direct effect	0.11 (−0.10, 0.32)	67%	0.19 (0.00, 0.38)	90%
Reference interaction	0.01 (−0.04, 0.05)	4%	−0.01 (−0.03, 0.02)	−3%
Mediated interaction	−0.01 (−0.09, 0.07)	−7%	0.01 (−0.02, 0.04)	4%
Pure indirect effect	0.06 (−0.01, 0.13)	36%	0.02 (−0.02, 0.06)	9%

CI, confidence interval.

Models were adjusted for TIDES center, maternal age, maternal race, income level, maternal education, parity, marital status, maternal smoking status, and stressful life events in the first trimester.

The strongest evidence of a sex-specific indirect effect was in the first trimester. In women carrying females, hCG operated through 2 of the 3 indirect effects that are estimated by this method. It operated through positive mediated interaction (74% of the total effect), and a negative pure indirect effect (−89% of the total effect). In women carrying males, there was evidence of a negative pure indirect effect of PAPPA that accounted for −39% of the total effect. There was a positive pure indirect effect of AFP in male fetuses in the second trimester (36% of the total effect, Table 3). Based on a comparison of the proportions, hCG was the strongest contributor to the total effect of all 5 placental-fetal biomarkers.

Based on the 2.5th and 97.5th percentiles generated in the bootstrapped analysis, the PIE was the single quantity with a meaningful statistical difference between male and female infants in the first trimester only (Table 4). The effect of hCG in mediating the maternal BMI association with birthweight was 0.08 units more negative in female vs. male infants. In 95% of simulations, the estimate of the difference in hCG mediation ranged from 0.21 units more negative in females vs. males to 0.02 units more positive in female vs. male infants. Conversely, the effect of PAPPA in mediating the maternal BMI association with birthweight was 0.09 units more positive in males vs. females. PAPPA mediation ranged from 0.20 units more positive in male vs. female infants to 0.01 units more negative in male vs. females (Table 4).

**Table 4:** Differences in the decomposition results between female and Male infants, for first trimester placental biomarkers only.

	Median difference (2.5th, 97.5th percentiles)	Median difference (2.5th, 97.5th percentiles)
	First trimester hCG *z*-score	First trimester PAPP-A *z*-score
Total effect	−0.22 (−0.55, 0.13)	−0.15 (−0.49, 0.18)
Controlled direct effect	−0.14 (−0.50, 0.21)	−0.21 (−0.56, 0.16)
Reference interaction	0.02 (−0.14, 0.19)	−0.06 (−0.27, 0.12)
Mediated interaction	−0.02 (−0.10, 0.06)	0.03 (−0.07, 0.14)
Pure indirect effect	−0.08 (−0.21, 0.02)	0.09 (−0.01, 0.20)

### Sensitivity analysis

This sensitivity analysis was conducted for hCG and PAPPA. The plausible bias in the TE, CDE, reference interaction effect, mediated interaction effect, and PIE when modeling hCG as a placental mediator respectively ranged within [−0.04, 0.01], [−0.05, 0.02], [−0.01, 0.002], [−0.003, 0.02], and [−0.01, 0.004] among male infants and ranges within [−0.05, 0.03], [−0.05, 0.02], [−0.004, 0.01], [−0.02, 0.01], and [−0.01, 0.02] among female infants respectively (Supplementary Tables S2A, S3A). For male infants, the signs or significance of the effects would remain unchanged if the bias were removed, indicating that the results are robust to omitted pre-exposure confounding (Supplementary Tables S2B, S3B).

The plausible bias in the TE, CDE, reference interaction effect, mediated interaction effect, and PIE when modeling PAPPA as a placental mediator respectively ranged within [−0.02, 0.05], [−0.03, 0.03], [−0.003, 0.007], [−0.01, 0.005], and [−0.003, 0.01] among male infants and ranges within [−0.04, 0.02], [−0.05, 0.02], [−0.001, 0.001], [−0.003, 0.003], and [−0.004, 0.003] among female infants respectively (Supplementary Tables S4A, S5A). For male and female infants, the signs or significance of the effects would remain unchanged if the bias were removed, indicating that the results were robust to omitted pre-exposure confounding (Supplementary Tables S4B, S5B).

## Discussion

Once decomposed, the association between maternal adiposity and birthweight, mediated by first trimester placental hCG and PAPPA, differed quantitatively and qualitatively between male and female infants. The overall effect of first trimester BMI on birthweight was positive and twice as strong in male vs. female infants. In male infants, PAPPA negatively mediated the association. In female infants, there was evidence of weak negative mediation by hCG and positive interaction of hCG and BMI in affecting birthweight. Future biomarker-based studies and/or functional studies using placental tissue are required to further elucidate these findings.

We applied here methods developed in the causal inference framework that require the investigator to pose sharper questions and offer explicit inferences on how these associations relate to a hypothetical intervention. At the same time, we do not want to overstate our findings, nor do we have strong enough evidence to propose a causal mechanism or a specific intervention. We use the language in the next paragraph, specified by the analytical method, to illustrate what a causal interpretation could look like, and what are the missing pieces that prohibit a causal interpretation.

These findings are interpreted as, if all women in this study at the 75th percentile of BMI (overweight) lost adequate weight, proportional to their height, to be at the 25th percentile (normal weight) before becoming pregnant, there would be a drop in birthweight equivalent to 20% of a standard deviation. According to the motivating hypothesis, this drop in birthweight would produce a reduction in the chronic disease burden (hypertension, diabetes, cardiovascular disease) as these children age. It is assumed that this decrease in BMI is occurring by ways of healthy diet and exercise, and not by way of smoking or other unhealthy practices that would cause weight reduction and negatively impact placental and fetal well-being (i.e., counterfactual consistency). In female infants, the effect of a decrease in maternal BMI on birthweight would be attenuated (approximately by 6%) by placental hCG. In male infants, the effect would be attenuated by 9% by PAPPA. The effect sizes of the indirect pathway were small in magnitude compared to the direct effects overall. This interpretation is predicated on satisfaction of causal assumptions (no unmeasured confounding, counterfactual consistency, positivity, no interference, no model misspecification), which are not fully achieved in this study (37). In terms of a biologic interpretation, the placenta-fetal unit, as measured by these 5 biomarkers, contributed weakly to the association of maternal BMI and infant birthweight. There may be other circulating placental biomarkers or tissue-level measures that would operate as stronger mediators.

The decomposition analysis has appeal as it allows for an interaction between the exposure and the mediator, which can also be a contributor to the mediation effect as reported here with hCG. However, the method does not allow for effect modification of the entire pathway (BMI > hCG > BW). We addressed this issue by stratifying by infant sex, and further evaluated through a bootstrap analysis of differences in the 5 quantities between males and females.

The 4-way decomposition analysis was strengthened by conducting a sensitivity analysis to assess the influence of unmeasured pre-exposure confounding. We obtained a plausible range of bias due to omitting a pre-exposure confounder that is comparable to the observed ones. In future research, a rigorous sensitivity analysis strategy can be developed for 4-way decomposition analysis by extending an intuitive simulation-based sensitivity analysis method for 2-way decomposition analysis (38). It will allow one to (1) test for conditional associations of an unmeasured pre-exposure confounder with the exposure, mediator, and outcome, (2) simulate the confounder from its conditional distribution, and (3) assess its influence by comparing the five effect estimates before and after adjusting for it in the analysis. In this study, we assume no post-exposure confounding of the mediator-outcome relationship as there are still few sensitivity analysis methods for assessing the influencing of post-exposure confounding (39–42).

hCG is a primary placental hormone of interest given its potential to mediate fetal endocrine disruption, a hypothesis elaborated on previously (10). First and second trimester hCG are associated with birthweight (11, 12, 43, 44); however, we are the first to report an inverse association of hCG and birthweight in female and male pregnancies. In this analysis, we included 4 additional placental-fetal biomarkers that are measured at the same time as hCG and are also available through the maternal medical record. These biomarkers were originally selected for prenatal screening due to their combined predictive value for fetal aneuploidies (26, 45). In all cases, they have also proven to be associated with fetal growth restriction and/or birthweight (11, 46–51). Pregnancy associated plasma protein (PAPPA) is a metalloproteinase that is produced primarily by the placenta in early pregnancy and promotes insulin growth factor (IGF) binding to its receptor (52). Low PAPPA levels in the first trimester are associated with higher risk of growth restriction (45). Estriol is a metabolite of estradiol expressed by the placenta as part of a regulatory mechanism to prevent virilization of female and feminization of male fetuses (53, 54). Low levels of estriol in the second trimester are associated with lower birthweight (47). Alpha fetoprotein (AFP) is a glycoprotein synthesized primarily by the embryo in early pregnancy and is secreted by the yolk sac, the fetal liver and the fetal gastrointestinal tract (55). The placenta is a minor source of AFP in early but does not produce AFP in late pregnancy (56, 57). Higher levels of alpha fetoprotein are associated with higher risk of growth restriction and low birthweight (46, 47). Finally, Inhibin-A is a glycoprotein that is synthesized by maternal, fetal and placental tissues (58). Higher levels of Inhibin-A are associated with lower birthweight and higher risk of growth restriction (50, 51).

We motivated inclusion of these analytes in the analysis as putative mediators given their associations with the outcome, and for the sake of comparison to the hCG association in the first trimester which is the primary association of interest. The lack of evidence of mediation by these additional analytes (other than first trimester PAPPA and second trimester AFP in male fetuses) generally supports the hypothesis that the placenta is more actively mediating or “programming” fetal development in the first trimester, and that these mechanisms by which maternal adiposity influences fetal growth are specific according to the sex of the fetus (19).

Strengths of this study are accurate measurement of individual-level biomarker and anthropometric data, comprehensive confounder data including maternal stress, the large number of male and female infants, the multi-center design, and the application of the 4-way decomposition with a sensitivity analysis. A limitation was relying on self-reported weight and height to calculate maternal BMI. Any reporting bias in the first trimester questionnaire would not likely be differential by placental biomarker levels or offspring birthweight. There was potential misspecification of the relationship when relying on a linear model. Based on scatter plots, there was not a pattern of a u-shape or an inverse u-shape in the associations presented here. With respect to external validity, the TIDES study population may not be representative of the overall US population given the high proportion of white women, and women with high income and graduate education.

Maternal urinary phthalates, an obesogenic endocrine disrupting chemical measured in this study, did not confound any of the associations reported here, and were not included in final models. Other obesogenic chemicals (Bisphenol A, organotins, perfluorooctanoic acid, etc.) may be unmeasured confounders (32).

In causal mediation analyses, there may be different sets of confounders for the exposure-outcome, the exposure-mediator, and the mediator-outcome relationships. The STATA med4way package used here only accommodates one set of confounders. This is a limitation; yet had minimal impact in the present analysis, as the two sets of confounders are essentially the same.

## Conclusions

In our example, the 4-way decomposition performed well in generating a specific hypothesis regarding the roles of hCG, PAPPA, and AFP in the sex-specific association of maternal adiposity and birthweight. To understand the health implications of this finding, it will be important to continue to study placental biomarkers as putative mediators of the pregnancy environment on child health. Decomposition can be a useful and accessible tool in biomarker-based epidemiology. We identified an overall association of maternal BMI and birthweight that was twice as strong in male vs. female infants. The discrepancy may be explained partly by a different mediation effect of the placenta in male vs. female pregnancies. This finding offers a new way of understanding and modeling the effects of maternal adiposity on fetal growth and birth size. Further studies are necessary to elucidate the biological mechanisms involving BMI, hCG and other placental biomarker levels, and fetal sex-specific responses in the early stages of pregnancy, to collect more information on sources of confounding and to more robustly interpret these findings in terms of fetal well-being.

## Data Availability

The data analyzed in this study is subject to the following licenses/restrictions: TIDES is a participating cohort in the Environmental Influences on Child Health Outcomes (ECHO) which has a process for requesting access to data. Requests to access these datasets should be directed to https://echochildren.org/.
