# Supplementary material for: Application of 4-way decomposition to the analysis of placental-fetal biomarkers as intermediary variables between maternal body mass index and birthweight
Source: Front Reprod Health. 2022 Dec 5;4:994436. doi: 10.3389/frph.2022.994436 (PMC9760955; doi:10.3389/frph.2022.994436)
Supplement: Supplementary file 1 [file Datasheet1.docx]

**Supplemental Table 1.** Comparison of the Characteristics of the first trimester prenatal screening subset and all subjects in The Infant Development and Environment Study (TIDES), 2010-2013.

|  | **Full TIDES**  N=753 | **Study subjects**  N=525 | **P-value** |
| --- | --- | --- | --- |
| **Maternal Age**, years, mean (SD) | 31.09 (5.52) | 31.38 (5.80) | 0.38 |
| **Maternal First Trimester BMI**, kg/m^2^, mean (SD) | 26.23 (6.29) | 26.37 (6.32) | 0.69 |
| **Birthweight,** kg, mean (SD) | 3.35 (0.56) | 3.33 (0.53) | 0.35 |
| **Female,** *N (%)* | 384 (51) | 250 (48) | 0.67 |
| **Race,** *N (%)* |  |  | 0.85 |
| White | 517 (69) | 350 (67) |  |
| Black | 91 (12) | 70 (13) |  |
| Asian | 49 (7) | 38 (7) |  |
| Other/Mixed | 92 (12) | 65 (12) |  |
| **Education,** *N (%)* |  |  | 0.18 |
| High school or less | 102 (14) | 87 (17) |  |
| Some college | 89 (12) | 72 (14) |  |
| College graduate | 235 (32) | 141 (27) |  |
| Graduate school | 319 (43) | 221 (42) |  |
| **Income,** *N (%)* |  |  | 0.19 |
| <$25,000 | 240 (33) | 172 (34) |  |
| $25,000-$74,999 | 131 (18) | 74 (15) |  |
| >=$75,000 | 356 (49) | 261 (51) |  |
| **Stress Events in the First Trimester,** *N (%)* |  |  | 0.48 |
| Any (>=1) | 146 (19) | 414 (79) |  |
| None | 607 (81) | 111 (21) |  |
| **Smoking Status,** *N (%)* |  |  | 0.18 |
| Not currently | 697 (94) | 478 (92) |  |
| Current | 43 (6) | 41 (8) |  |
| **Parity,** *N (%)* |  |  | 0.41 |
| 0 | 277 (38) | 176 (34) |  |
| 1 | 212 (29) | 155 (30) |  |
| >1 | 238 (33) | 180 (35) |  |
| **Marital Status,** *N (%)* |  |  | 0.16 |
| Partnered | 622 (83) | 418 (80) |  |
| Single/ Previously Married | 126 (17) | 105 (20) |  |
| **Assisted Reproductive Technology,** *N (%)* |  |  | 0.41 |
| No | 707 (94) | 486 (93) |  |
| Yes | 46 (6) | 486 (93) |  |
| **Preterm Birth,** *N (%)* |  |  | 1.00 |
| No | 684 (91) | 478 (91) |  |
| Yes | 67 (9) | 47 (9) |  |

**Supplemental Table 2a.** Sensitivity analysis for first trimester hCG in women carrying male fetuses: bias in each effect estimate due to removal of each observed pre-exposure covariate in the original model.

|  | Original | Bias Due to Removal of | | | | | | | | |
| --- | --- | --- | --- | --- | --- | --- | --- | --- | --- | --- |
|  |  | Stressful life events | Parity | Marital status | Income | Race | Education level | Maternal age | Center | Smoke |
| Total effect | 0.27  (0.05, 0.50) | -0.0064 | 0.0069 | 0.0027 | -0.0288 | 0.0144 | 0.0034 | -0.0224 | -0.0385 | -0.0056 |
| Controlled direct effect | 0.23  (-0.01, 0.46) | -0.0041 | 0.0118 | 0.0073 | -0.0292 | 0.0126 | 0.0022 | -0.0227 | -0.0375 | -0.0040 |
| Reference interaction | -0.03  (-0.08, 0.03) | -0.0014 | 0.0062 | 0.0021 | -0.0105 | 0.0016 | -0.0025 | 0.0009 | -0.0001 | -0.0023 |
| Mediated interaction | 0.05  (-0.04, 0.14) | 0.0027 | -0.0128 | -0.0039 | 0.0211 | -0.0029 | 0.0045 | -0.0017 | 0.0000 | 0.0022 |
| Pure indirect effect | 0.03  (-0.04, 0.10) | -0.0036 | 0.0017 | -0.0028 | -0.0101 | 0.0030 | -0.0009 | 0.0011 | -0.0010 | -0.0015 |

**Supplemental Table 2b.** Sensitivity analysis for first trimester hCG in women carrying male fetuses: adjusted point estimate and confidence interval with adjustment for an unmeasured pre-exposure covariate whose confounding role is comparable to each observed pre-exposure covariate. Bold indicates the confidence interval does not include the null value.

|  | Original | *Results after the removal of a bias associated with an unmeasured pre-exposure covariate whose confounding role is comparable to | | | | | | | | |
| --- | --- | --- | --- | --- | --- | --- | --- | --- | --- | --- |
|  |  | Stressful life events | Parity | Marital status | Income | Race | Education level | Maternal age | Center | Smoke |
| Total effect | 0.27  (0.05, 0.50) | 0.27  (0.05, 0.49) | 0.28  (0.07, 0.49) | 0.28  (0.06, 0.50) | 0.25  (0.02, 0.47) | 0.29  (0.06, 0.51) | 0.28  (0.06, 0.50) | 0.25  (0.03, 0.47) | 0.24  (0.02, 0.45) | 0.27  (0.05, 0.49) |
| Controlled direct effect | 0.23  (-0.01, 0.46) | 0.22  (-0.01, 0.46) | 0.24  (0.02, 0.46) | 0.23  (0.00, 0.46) | 0.20  (-0.04, 0.43) | 0.24  (0.00, 0.47) | 0.23  (0.00, 0.46) | 0.20  (-0.03, 0.44) | 0.19  (-0.04, 0.41) | 0.22  (-0.01, 0.45) |
| Reference interaction | -0.03  (-0.08, 0.03) | -0.03  (-0.08, 0.03) | -0.02  (-0.07, 0.03) | -0.02  (-0.07, 0.03) | -0.04  (-0.09, 0.02) | -0.02  (-0.08, 0.03) | -0.03  (-0.08, 0.02) | -0.02  (-0.08, 0.03) | -0.03  (-0.08, 0.02) | -0.03  (-0.08, 0.03) |
| Mediated interaction | 0.05  (-0.04, 0.14) | 0.05  (-0.05, 0.15) | 0.03  (-0.05, 0.11) | 0.04  (-0.04, 0.13) | 0.07  (-0.02, 0.16) | 0.04  (-0.05, 0.14) | 0.05  (-0.04, 0.14) | 0.04  (-0.04, 0.13) | 0.05  (-0.04, 0.13) | 0.05  (-0.04, 0.14) |
| Pure indirect effect | 0.03  (-0.04, 0.10) | 0.02  (-0.05, 0.10) | 0.03  (-0.04, 0.10) | 0.02  (-0.04, 0.09) | 0.02  (-0.05, 0.08) | 0.03  (-0.04, 0.10) | 0.03  (-0.04, 0.09) | 0.03  (-0.04, 0.10) | 0.03  (-0.04, 0.09) | 0.03  (-0.04, 0.09) |

*Adjusted effect estimate = original estimate – bias

**Supplemental Table 3a.** Sensitivity analysis for first trimester hCG in women carrying female fetuses: bias in each effect estimate due to removal of each observed pre-exposure covariate in the original model.

|  | Original | Bias Due to Removal of | | | | | | | | |
| --- | --- | --- | --- | --- | --- | --- | --- | --- | --- | --- |
|  |  | Stressful life events | Parity | Marital status | Income | Race | Education level | Maternal age | Center | Smoke |
| Total effect | 0.08  (-0.10, 0.27) | 0.0095 | -0.0168 | 0.0069 | -0.0677 | -0.0198 | -0.0123 | -0.0550 | -0.0090 | 0.0291 |
| Controlled direct effect | 0.11  (-0.08, 0.30) | 0.0126 | -0.0157 | 0.0064 | -0.0634 | -0.0161 | -0.0115 | -0.0615 | -0.0119 | 0.0282 |
| Reference interaction | -0.04  (-0.09, 0.01) | 0.0140 | 0.0035 | 0.0008 | 0.0030 | -0.0034 | -0.0025 | 0.0068 | 0.0001 | 0.0027 |
| Mediated interaction | 0.06  (0.00, 0.13) | -0.0233 | -0.0041 | -0.0013 | -0.0064 | 0.0056 | 0.0042 | -0.0112 | -0.0001 | -0.0031 |
| Pure indirect effect | -0.06  (-0.12, 0.01) | 0.0061 | -0.0004 | 0.0010 | -0.0010 | -0.0059 | -0.0024 | 0.0110 | 0.0029 | 0.0014 |

**Supplemental Table 3b.** Sensitivity analysis for first trimester hCG in women carrying female fetuses: adjusted point estimate and confidence interval with adjustment for an unmeasured pre-exposure covariate whose confounding role is comparable to each observed pre-exposure covariate. Bold indicates the confidence interval does not include the null value.

|  | Original | *Results after the removal of a bias associated with an unmeasured pre-exposure covariate whose confounding role is comparable to | | | | | | | | |
| --- | --- | --- | --- | --- | --- | --- | --- | --- | --- | --- |
|  |  | Stressful life events | Parity | Marital status | Income | Race | Education level | Maternal age | Center | Smoke |
| Total effect | 0.08  (-0.10, 0.27) | 0.09  (-0.10, 0.28) | 0.07  (-0.11, 0.24) | 0.09  (-0.09, 0.27) | 0.01  (-0.16, 0.19) | 0.06  (-0.12, 0.24) | 0.07  (-0.11, 0.25) | 0.03  (-0.15, 0.21) | 0.07  (-0.11, 0.26) | 0.11  (-0.06, 0.28) |
| Controlled direct effect | 0.11  (-0.08, 0.30) | 0.12  (-0.07, 0.32) | 0.10  (-0.09, 0.28) | 0.12  (-0.07, 0.30) | 0.05  (-0.13, 0.23) | 0.10  (-0.09, 0.28) | 0.10  (-0.09, 0.29) | 0.05  (-0.13, 0.23) | 0.10  (-0.09, 0.29) | 0.14  (-0.04, 0.32) |
| Reference interaction | -0.04  (-0.09, 0.01) | -0.03  (-0.07, 0.02) | -0.04  (-0.08, 0.01) | -0.04  (-0.08, 0.01) | -0.04  (-0.08, 0.01) | -0.04  (-0.09, 0.01) | -0.04  (-0.09, 0.01) | -0.03  (-0.07, 0.01) | -0.04  (-0.09, 0.01) | -0.04  (-0.08, 0.01) |
| Mediated interaction | 0.06  (0.00, 0.13) | 0.04  (-0.02, 0.10) | 0.06  (0.00, 0.13) | 0.06  (0.00, 0.13) | 0.06  (-0.01, 0.12) | 0.07  (0.00, 0.14) | 0.07  (0.00, 0.14) | 0.05  (-0.01, 0.11) | 0.07  (0.00, 0.13) | 0.06  (0.00, 0.12) |
| Pure indirect effect | -0.06  (-0.12, 0.01) | -0.05  (-0.11, 0.01) | -0.06  (-0.12, 0.01) | -0.05  (-0.12, 0.01) | -0.06  (-0.12, 0.01) | -0.06  (-0.13, 0.00) | -0.06  (-0.12, 0.01) | -0.04  (-0.10, 0.01) | -0.05  (-0.11, 0.01) | -0.05  (-0.11, 0.01) |

*Adjusted effect estimate = original estimate – bias

**Supplemental Table 4a.** Sensitivity analysis for first trimester PAPP-A in women carrying male fetuses: bias in each effect estimate due to removal of each observed pre-exposure covariate in the original model.

|  | Original | Bias Due to Removal of | | | | | | | | |
| --- | --- | --- | --- | --- | --- | --- | --- | --- | --- | --- |
|  |  | Stressful life events | Parity | Marital status | Income | Race | Education level | Maternal age | Center | Smoke |
| Total effect | 0.23  (0.01, 0.45) | -0.0034 | 0.0513 | 0.0009 | -0.0230 | 0.0245 | 0.0019 | -0.0039 | -0.0073 | -0.0014 |
| Controlled direct effect | 0.29  (0.05, 0.52) | -0.0053 | 0.0452 | 0.0006 | -0.0287 | 0.0282 | 0.0015 | -0.0050 | -0.0123 | -0.0048 |
| Reference interaction | -0.04  (-0.12, 0.04) | 0.0020 | 0.0055 | -0.0005 | 0.0016 | 0.0016 | -0.0007 | 0.0070 | -0.0026 | 0.0020 |
| Mediated interaction | 0.07  (-0.07, 0.21) | -0.0035 | -0.0133 | 0.0009 | -0.0014 | -0.0028 | 0.0012 | -0.0123 | 0.0046 | -0.0022 |
| Pure indirect effect | -0.09  (-0.17, 0.00) | 0.0034 | 0.0139 | -0.0001 | 0.0054 | -0.0025 | -0.0001 | 0.0065 | 0.0030 | 0.0036 |

**Supplemental Table 4b.** Sensitivity analysis for first trimester PAPP-A in women carrying male fetuses: adjusted point estimate and confidence interval with adjustment for an unmeasured pre-exposure covariate whose confounding role is comparable to each observed pre-exposure covariate. Bold indicates the confidence interval does not include the null value.

|  | Original | *Results after the removal of a bias associated with an unmeasured pre-exposure covariate whose confounding role is comparable to | | | | | | | | |
| --- | --- | --- | --- | --- | --- | --- | --- | --- | --- | --- |
|  |  | Stressful life events | Parity | Marital status | Income | Race | Education level | Maternal age | Center | Smoke |
| Total effect | 0.23  (0.01, 0.45) | 0.23  (0.01, 0.44) | 0.28  (0.07, 0.49) | 0.23  (0.01, 0.45) | 0.21  (-0.02, 0.43) | 0.25  (0.03, 0.47) | 0.23  (0.01, 0.45) | 0.22  (0.01, 0.44) | 0.22  (0.01, 0.44) | 0.23  (0.01, 0.44) |
| Controlled direct effect | 0.29  (0.05, 0.52) | 0.28  (0.04, 0.52) | 0.33  (0.11, 0.56) | 0.29  (0.05, 0.53) | 0.26  (0.01, 0.50) | 0.31  (0.07, 0.55) | 0.29  (0.05, 0.53) | 0.28  (0.04, 0.52) | 0.27  (0.04, 0.51) | 0.28  (0.04, 0.52) |
| Reference interaction | -0.04  (-0.12, 0.04) | -0.04  (-0.12, 0.04) | -0.03  (-0.12, 0.05) | -0.04  (-0.12, 0.04) | -0.04  (-0.12, 0.04) | -0.04  (-0.12, 0.05) | -0.04  (-0.12, 0.04) | -0.03  (-0.11, 0.05) | -0.04  (-0.12, 0.04) | -0.04  (-0.12, 0.04) |
| Mediated interaction | 0.07  (-0.07, 0.21) | 0.07  (-0.07, 0.21) | 0.06  (-0.08, 0.19) | 0.07  (-0.07, 0.21) | 0.07  (-0.07, 0.21) | 0.07  (-0.08, 0.21) | 0.07  (-0.07, 0.21) | 0.06  (-0.08, 0.20) | 0.08  (-0.06, 0.22) | 0.07  (-0.07, 0.21) |
| Pure indirect effect | -0.09  (-0.17, 0.00) | -0.09  (-0.17, 0.00) | -0.07  (-0.16, 0.01) | -0.09  (-0.17, 0.00) | -0.08  (-0.17, 0.00) | -0.09  (-0.18, -0.01) | -0.09  (-0.17, -0.01) | -0.08  (-0.16, 0.00) | -0.09  (-0.17, 0.00) | -0.08  (-0.17, 0.00) |

*Adjusted effect estimate = original estimate – bias

**Supplemental Table 5a.** Sensitivity analysis for first trimester PAPP-A in women carrying female fetuses: bias in each effect estimate due to removal of each observed pre-exposure covariate in the original model.

|  | Original | Bias Due to Removal of | | | | | | | | |
| --- | --- | --- | --- | --- | --- | --- | --- | --- | --- | --- |
|  |  | Stressful life events | Parity | Marital status | Income | Race | Education level | Maternal age | Center | Smoke |
| Total effect | 0.10  (-0.06, 0.26) | -0.0109 | -0.0023 | 0.0043 | -0.0354 | -0.0106 | -0.0155 | -0.0426 | -0.0099 | 0.0219 |
| Controlled direct effect | 0.10  (-0.06, 0.26) | -0.0046 | -0.0043 | 0.0047 | -0.0381 | -0.0076 | -0.0191 | -0.0456 | -0.0091 | 0.0230 |
| Reference interaction | -0.01  (-0.04, 0.02) | -0.0031 | -0.0009 | 0.0007 | 0.0012 | -0.0011 | 0.0016 | 0.0001 | -0.0005 | 0.0007 |
| Mediated interaction | 0.02  (-0.02, 0.06) | 0.0046 | 0.0025 | -0.0012 | -0.0021 | 0.0017 | -0.0025 | -0.0004 | 0.0007 | -0.0009 |
| Pure indirect effect | -0.01  (-0.05, 0.03) | -0.0079 | 0.0004 | 0.0001 | 0.0037 | -0.0036 | 0.0044 | 0.0034 | -0.0009 | -0.0008 |

**Supplemental Table 5b.** Sensitivity analysis for first trimester PAPP-A in women carrying female fetuses: adjusted point estimate and confidence interval with adjustment for an unmeasured pre-exposure covariate whose confounding role is comparable to each observed pre-exposure covariate. Bold indicates the confidence interval does not include the null value.

|  | Original | *Results after the removal of a bias associated with an unmeasured pre-exposure covariate whose confounding role is comparable to | | | | | | | | |
| --- | --- | --- | --- | --- | --- | --- | --- | --- | --- | --- |
|  |  | Stressful life events | Parity | Marital status | Income | Race | Education level | Maternal age | Center | Smoke |
| Total effect | 0.10  (-0.06, 0.26) | 0.09  (-0.07, 0.25) | 0.10  (-0.06, 0.25) | 0.10  (-0.05, 0.26) | 0.06  (-0.08, 0.21) | 0.09  (-0.07, 0.24) | 0.08  (-0.07, 0.24) | 0.06  (-0.09, 0.21) | 0.09  (-0.07, 0.24) | 0.12  (-0.03, 0.27) |
| Controlled direct effect | 0.10  (-0.06, 0.26) | 0.10  (-0.06, 0.26) | 0.10  (-0.06, 0.26) | 0.11  (-0.05, 0.26) | 0.06  (-0.09, 0.21) | 0.10  (-0.06, 0.25) | 0.08  (-0.07, 0.24) | 0.06  (-0.10, 0.21) | 0.09  (-0.06, 0.25) | 0.13  (-0.03, 0.28) |
| Reference interaction | -0.01  (-0.04, 0.02) | -0.01  (-0.04, 0.02) | -0.01  (-0.04, 0.02) | -0.01  (-0.04, 0.02) | -0.01  (-0.04, 0.02) | -0.01  (-0.04, 0.02) | -0.01  (-0.04, 0.02) | -0.01  (-0.04, 0.01) | -0.01  (-0.04, 0.02) | -0.01  (-0.04, 0.01) |
| Mediated interaction | 0.02  (-0.02, 0.06) | 0.02  (-0.02, 0.06) | 0.02  (-0.02, 0.06) | 0.02  (-0.02, 0.05) | 0.01  (-0.02, 0.05) | 0.02  (-0.02, 0.06) | 0.01  (-0.02, 0.05) | 0.02  (-0.02, 0.05) | 0.02  (-0.02, 0.06) | 0.02  (-0.02, 0.05) |
| Pure indirect effect | -0.01  (-0.05, 0.03) | -0.02  (-0.06, 0.03) | -0.01  (-0.05, 0.03) | -0.01  (-0.05, 0.03) | -0.01  (-0.04, 0.03) | -0.01  (-0.05, 0.03) | 0.00  (-0.04, 0.03) | -0.01  (-0.04, 0.03) | -0.01  (-0.05, 0.03) | -0.01  (-0.05, 0.03) |

*Adjusted effect estimate = original estimate – bias

**
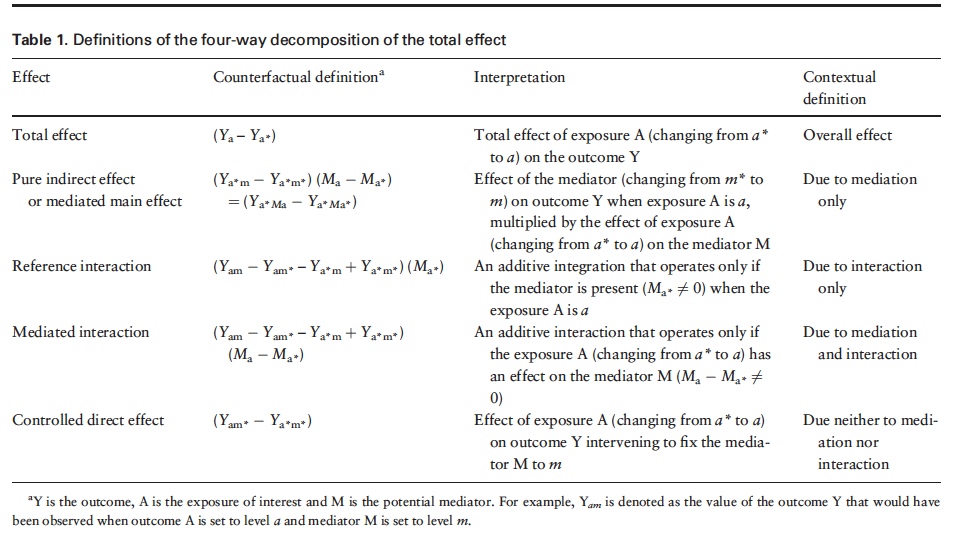
**Supplemental Figure 1. Description of the quantities calculated in the 4-way decomposition, excerpted from Lee et al. 2018. IJE*.*
